# All Paired Up with No Place to Go: Pairing, Synapsis, and DSB Formation in a Balancer Heterozygote

**DOI:** 10.1371/journal.pgen.0010067

**Published:** 2005-11-18

**Authors:** Wei J Gong, Kim S McKim, R. Scott Hawley

**Affiliations:** 1 Stowers Institute for Medical Research, Kansas City, Missouri, United States of America; 2 Waksman Institute and Department of Genetics, Rutgers, the State University of New Jersey, Piscataway, New Jersey, United States of America; 3 Department of Molecular Biosciences, University of Kansas, Lawrence, Kansas, United States of America; The Jackson Laboratory, United States of America

## Abstract

The multiply inverted *X* chromosome balancer *FM7* strongly suppresses, or eliminates, the occurrence of crossing over when heterozygous with a normal sequence homolog. We have utilized the LacI-GFP: *lacO* system to visualize the effects of *FM7* on meiotic pairing, synapsis, and double-strand break formation in *Drosophila* oocytes. Surprisingly, the analysis of meiotic pairing and synapsis for three *lacO* reporter couplets in *FM7/X* heterozygotes revealed they are paired and synapsed during zygotene/pachytene in 70%–80% of oocytes. Moreover, the regions defined by these *lacO* couplets undergo double-strand break formation at normal frequency. Thus, even complex aberration heterozygotes usually allow high frequencies of meiotic pairing, synapsis, and double-strand break formation in *Drosophila* oocytes. However, the frequencies of failed pairing and synapsis were still 1.5- to 2-fold higher than were observed for corresponding regions in oocytes with two normal sequence *X* chromosomes, and this effect was greatest near a breakpoint. We propose that heterozygosity for breakpoints creates a local alteration in synaptonemal complex structure that is propagated across long regions of the bivalent in a fashion analogous to chiasma interference, which also acts to suppress crossing over.

## Synopsis

One of the more intriguing mysteries in chromosome biology lies in the ability of homologous chromosomes to pair during meiosis, the process that creates haploid gametes. This pairing is the crucial first step in seeing to it that each gamete receives one, and only one, copy of each chromosome. The later steps in this process include recombination and the actual segregation of paired homologs into different daughter cells. During the last century of study, people who worked on meiosis believed that changes in chromosome structure that disrupted the meiotic processes did so by impeding the pairing process. Here the authors show that pairing occurs quite normally even in cells carrying a highly rearranged chromosome. Surprisingly, even recombination is normally initiated, but not completed. These data are allowing them to reconsider several old and cherished views of the process called meiosis.

## Introduction

Despite recent advances in our understanding of the meiotic process, the mechanisms that underlie meiotic pairing and the establishment of synapsis remain poorly understood. This is particularly true for *Drosophila* female meiosis, both because the earlier stages of female meiosis are rapid and therefore difficult to analyze by standard cytogenetic techniques and because of the paucity of mutants that affect the pairing process. Recently, Sherizen et al. [1] in *Drosophila* females and Vazquez et al. [2] in *Drosophila* males have presented evidence that meiotic pairings in *Drosophila* could be an extension of existing pre-meiotic pairings. In other words, the pairing events that take place in cycles 14–15 of *Drosophila* embryos [3] could be maintained throughout germline differentiation and development, without necessitating a period of re-pairing in meiotic prophase. This observation supports the assertion made by Roeder and Weiner et al. [4,5] that the ability of *Drosophila* females to form a synaptonemal complex (SC) between homologous chromosomes in the absence of double-strand breaks (DSBs) [6] reflects the fact that these chromosomes enter meiosis as paired.

However, the suggestion that meiotic pairing is a continuation of pre-existing somatic pairings assumes that somatic pairings are maintained through the different phases of the cell cycle. In fact, there are notable examples where somatic pairing is lost in *Drosophila* somatic cells. For example, although Vazquez et al. [2] suggested that somatic pairing was maintained through pre-meiotic S-phase in the male germline, homolog pairing is reduced or lost during S-phase in larval neuroblasts [7] and during anaphase in embryos [8]. These observations suggest that both meiotic and mitotic pairings might need to be re-established, perhaps more than once, during each cell cycle. Thus, it is possible that despite previous somatic pairings, pairing might still need to be re-established in female meiotic prophase immediately prior to SC formation. In other words, rather than proposing that meiotic pairings are extensions of somatic pairings, it is possible that homolog pairing during prophase I in *Drosophila* oocytes could occur by an efficient and rapid mechanism that functions in somatic cells as well.

For obvious reasons then, the study of meiotic pairing in *Drosophila* must be re-phrased in terms of three distinct sets of questions. First, how do the somatic pairings that occur in *Drosophila* embryonic cells and in other tissues take place? Second, are those pairings maintained through division and development? Third, regardless of pre-existing somatic pairings, how does the meiotic cell facilitate meiotic synapsis and recombination? In this paper we address the third of these questions by investigating how heterozygosity for structurally altered homologs affects the maintenance of meiotic pairing, the assembly of the SC, and the initiation of genetic recombination.

In *Drosophila,* and in most other higher organisms, heterozygous chromosome aberrations act as dominant and region-specific suppressors of meiotic crossing over [9,10]. It has been reasonably assumed these exchange suppressions result from defects in either meiotic pairing or synapsis. However, studies of translocation heterozygotes in tomato by Herickhoff et al. [11] and more recently in *Drosophila* by Sherizen et al. [1] have shown that pairing and synapsis occur normally in translocation heterozygotes, even in regions close to the breakpoint, suggesting that the processes that mediate pairing and synapsis in higher eukaryotes may be insensitive to breakpoint heterozygosity. In order to examine the effects of more severe structural rearrangement on pairing, synapsis, and exchange, we focused on the effects of a multiply inverted balancer chromosome that, when heterozygous, suppresses exchange along the entire chromosome.

**Figure 1:**
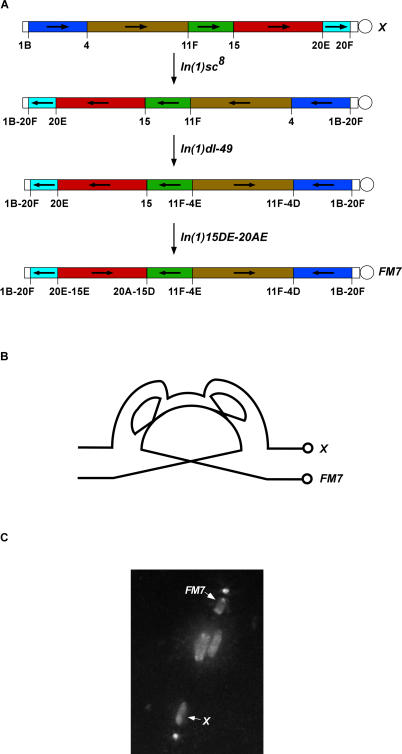
The Multiply Inverted Balancer Chromosome *FM7* (A) Schematic diagram of the generation of chromosomal inversions from *X* to *FM7*. Color represents the regions that are involved in inversions, and arrows are used to indicate the orientation of each region with respect to the centromere. Circles represent the centromeres. The numbers below each structure represent positions of the standard polytene map. (B) A hypothetical structure displaying the pairing relations of the two homologs in an *FM7/X* heterozygote. (C) A picture of DAPI-stained chromosome at meiotic metaphase I in *FM7/X* oocytes (courtesy of W. Gilliland). The two *X* and 4th chromosomes are positioned between the two autosomal bivalents and the poles, with the dot-like 4th chromosomes located closer to the poles. The *FM7* chromosome (denoted by the brighter DAPI staining at its tip) is located between the autosomes and the upper pole, while the normal sequence *X* chromosome lies in the lower half of the spindle. Note that the *X* and *FM7* chromosomes are not connected by chiasmata.

Specifically, we set out to examine the effects of an *X* chromosome balancer known as *FM7* [12]. As shown in Figure 1A, the *FM7* chromosome differs from a normal sequence homolog by three separate but overlapping paracentric inversions: a large inversion, *(In(1)sc^8^),* that spans the length of the *X* chromosome, and two smaller inversions, *(In(1)dl-49* and *In(1)15DE-20AE),* that both lie within *In(1)sc^8^*. Thus, *FM7* differs in sequence from a normal sequence *X* chromosome by six breakpoints distributed along the length of the *X* chromosome, four of which (1B, 4D, 11F, and 15DE) disrupt the euchromatin. The remaining two breaks (20A and 20E) disrupt the centric heterochromatin. The complex juxtaposition of homologs that would be required to fully pair both *X* chromosomes in *FM7/X* heterozygotes is displayed in Figure 1B.

Heterozygosity for *FM7* results in a complete, or near complete, suppression of exchange along the length of the *X* chromosome. Three lines of evidence demonstrate that heterozygosity for *FM7* actually suppresses the formation of crossover products and does not simply prevent or preclude their recovery, as is characteristic of many large paracentric inversions [13,14]. First, as exemplified in Figure 1C, a cytological analysis of metaphase I figures in *FM7/X* heterozygotes by Theurkauf and Hawley [15] revealed that the *X* chromosomes were usually, if not always, achiasmate. Indeed, in 28/28 metaphase figures observed by Theurkauf and Hawley [15], both the *X* and *FM7* chromosomes were always observed as well separated elements, such that one *X* chromosome was positioned between one pole and the chiasmate chromosomes while the other *X* chromosome was observed at a symmetrical position on the other half of the spindle. These observations parallel the behavior of the obligately achiasmate *4th* chromosome.

Second, the segregation of the *X* chromosomes in *FM7/X* heterozygotes is entirely dependent on the functioning of the achiasmate-specific distributive system [16–19]. In *Drosophila* females that are homozygous or hemizygous for null mutants that specifically ablate the achiasmate segregation system, such as *ald, alpha-Tub67C, nod,* and *mtrm*, the frequency of *X* nondisjunction in *FM7/X* females approaches 50%, the value expected for random segregation if all, or nearly all, oocytes are achiasmate with respect to the *X* chromosomes [16–19].

Finally, the use of sophisticated genetic regimes by Novitski and Braver [10] to recover crossovers that do occur within females heterozygous only for the *In(1)dl-49* chromosome demonstrated that exchange within this single inversion is reduced to less than 25% of normal and exchange is also strongly suppressed immediately distal to this inversion. The imposition of the remaining two inversions that make up the *FM7* chromosome is expected to further reduce or eliminate even these residual levels of exchange. Indeed, Hutter [20] has attempted to recover rare exchanges within the proximal *In(1)15DE;20AE* inversion of *FM7/X* heterozygotes and estimates that if such exchanges occur at all, they do so at frequencies less than 10%–20% of what might be expected based on the genetic length of this interval. These studies all argue that, when heterozygous, the *FM7* chromosome functions to suppress crossing over, rather than simply eliminating crossovers that do occur [13,14]. This cannot be due to any direct effect of the *FM7* chromosome, the genes it carries, or the process of crossing over itself, because crossing over in *FM7* homozygotes is normal [16].

Despite extensive study of its effects on recombination, very little is known about the effects of heterozygosity for *FM7* (or of any balancer chromosome) on meiotic pairing and synapsis in *Drosophila* oocytes. Dernburg et al. [21] showed that the blocks of homologous heterochromatin remained paired throughout meiotic prophase in *FM7/X* heterozygotes. This observation forms the linchpin of the model that shows that achiasmate segregations are mediated by the maintenance of heterochromatic pairing, but no data on the pairing or synapsis of euchromatic regions are available. To assess pairing in oocytes, we took advantage of the LacI-GFP: *lacO* system developed by Robinett et al. [22] and applied it to the *Drosophila* male meiotic system by Vazquez et al. [2]. In this system, specific sites on a given pair of homologs are marked by the insertion of an array of *lacO* binding sites. Expression of the LacI-GFP fusion protein under the control of a germline-specific promoter (in this case, the *nanos* promoter) results in the binding of LacI-GFP to the array of *lacO* sites and thus allows the pairing of a given site to be assayed as unpaired or paired.

Surprisingly, our analysis revealed a high frequency of pairing and synapsis in *FM7/X* females. Moreover, at least at the level of light microscopy, the pairing and synapsis we observe is similar to that observed in females carrying two structurally normal *X* chromosomes. Thus, the strong reduction of recombination observed in these oocytes cannot be accounted for by a correspondingly strong defect in pairing and synapsis. We also observed a normal frequency of DSB formation on the *X* chromosomes in *FM7/X* heterozygotes, suggesting that the events that initiate meiotic recombination occur normally and are not impeded by structural heterozygosity. Indeed, the frequency of DSBs per oocyte is unchanged even in oocytes that are heterozygotes for balancers that suppress exchange on all three chromosomes.

## Results

### Use of the LacI-GFP: *lacO* System to Assess Chromosome Pairing and Synapsis in *Drosophila* Oocytes with Two Normal Sequence *X* Chromosomes

We set out to assess pairing and synapsis in *Drosophila* females that either carry two normal sequence *X* chromosomes or are heterozygous for a normal sequence *X* chromosome and for the multiply inverted *FM7* chromosome [12]. To obtain *lacO* sites in corresponding positions on both the *X* chromosome and the *FM7* balancer chromosome, we mobilized a *lacO* array located on Chromosome 2 to multiple sites on both the normal sequence *X* and *FM7* and mapped the positions of these insertions on the *X* chromosome genomic sequence by inverse PCR.

**Figure 2:**
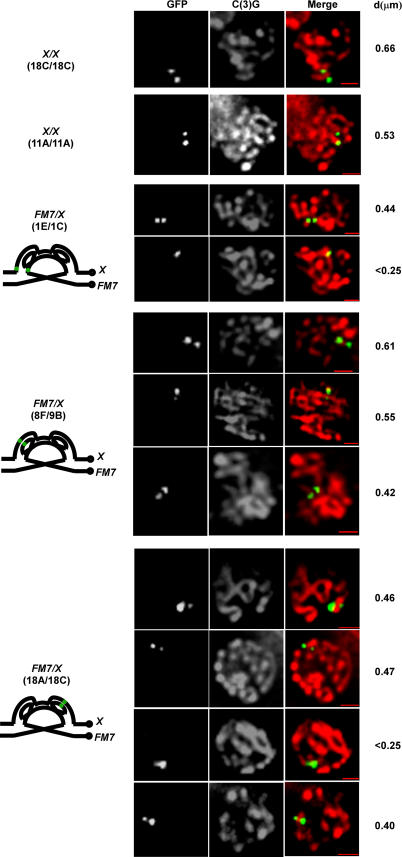
Synapsed *lacO* Foci Flanking Stretches of SC in *FM7/X* or *X/X* Oocytes In both *X/X* and *FM7/X* oocytes, paired *lacO* foci flank regions of SC. The positions of paired/synapsed *lacO* sites studied in *X/X* and *FM7/X* oocytes in zygotene/pachytene are shown at the left-most of each row. One to three optical sections show two partially overlapping or adjacent GFP foci (green) associated with a segment of SC (red). Distances between those GFP foci are shown at the right-most in each row. Due to the difficulty of accurately measuring the distance between overlapping foci, “<0.25” is used. Bars = 1 μm.

**Figure 3:**
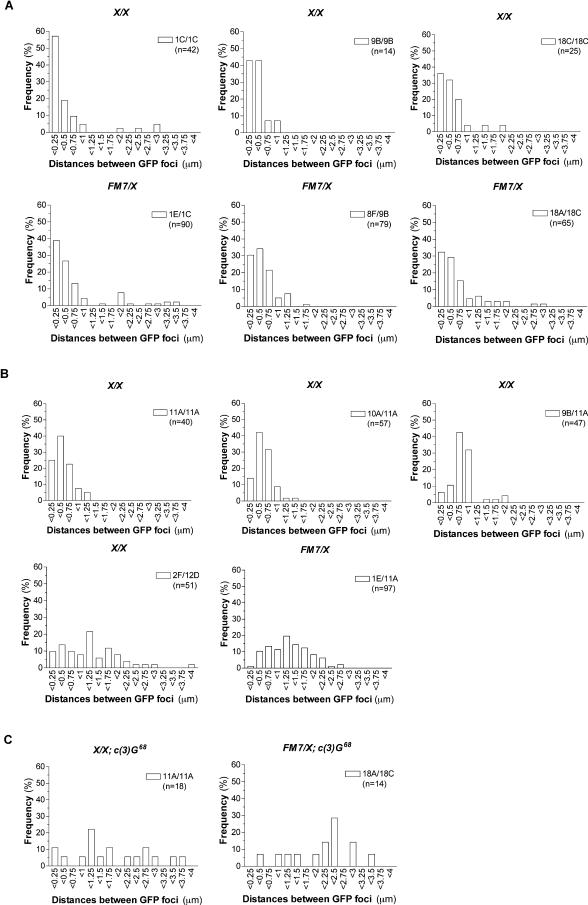
Distributions of Distances between GFP Foci in Both *X/X* and *FM7/X* Oocytes These data report, in histogram form, the distribution of distances between GFP foci in those oocytes with two distinct GFP foci (including those oocytes with overlapping foci). For those oocytes with overlapping foci, the distance is measured as < 0.25 μm. (A) The distribution of distances in oocytes containing two *lac*O insertions at allelic sites in *X/X* females (upper panel) and nearby *lacO* couplets in *FM7/X* oocytes (lower panel). (B) The distribution of distances for non-allelic lacO insertion sites in *X/X* and *FM7/X* females. The distribution for an allelic pair of lacO insertions at 11A is provided as a control. (C) The distribution of distances in oocytes containing two *lac*O insertions at an allelic site (11A) in *X/X; c(3)G* females (left) and a nearby *lacO* couplet (18A/18C) in *FM7/X ; c(3)G* oocytes (right panel).

Our initial analysis of chromosome pairing and synapsis in *Drosophila* oocytes focused on the study of four allelic pairs of *lacO* arrays located at 1C, 9B, 11A, and 18C on a pair of normal sequence *X* chromosomes (Table 1 and Figures 2 and 3). In SC-positive oocytes the two *lacO* sites are considered as paired and synapsed if any of the following three criteria are met: 1) there is only one visible green fluorescent protein (GFP) focus associated with a stretch of SC; 2) there are two clearly overlapping GFP foci associated with a stretch of SC (see the penultimate row in Figure 2); or 3) there are two distinct GFP foci that lie on opposite sides of a stretch of SC (see Figure 2). Using this method, the observed frequencies of failed synapsis for the four allelic pairs of *lacO* insertions studied in *X*/*X* oocytes ranged from 1.7% for the *lacO* insertion at 9B, to values ranging from 4.2%–4.6% for the *lacO* insertions at 1C, 11A, and 18C.

**Table 1:**
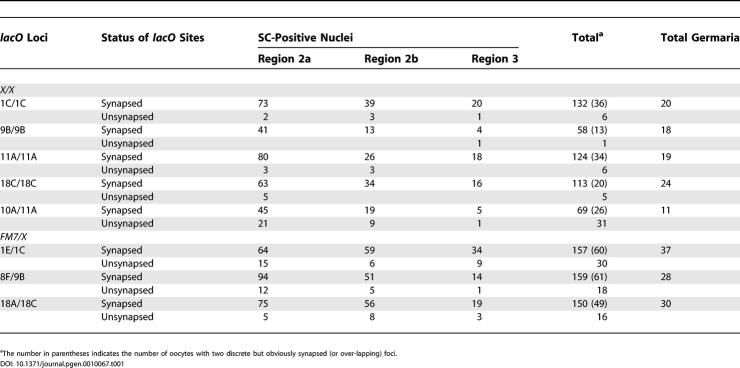
Chromosome Synapsis As Assayed by LacI-GFP Tagging

However, as noted in the Materials and Methods, on average any given *lacO* array was detectable in only 70% of the oocytes, and thus two well-separated *lacO* arrays would be detectable in only approximately 50% of the oocytes in which they occurred. This required us to use two additional metrics to estimate the frequency of failed pairing and synapsis. First, we provide a more accurate measurement of pairing/synapsis failure by multiplying the observed fraction of oocytes with unpaired *lacO* foci by a factor of two. Second, we obviate the detection problem by considering only that subset of oocytes that exhibit two discernable foci. In Table 1, the number of synapsed GFP foci that were discernable as two distinct or overlapping dots flanking the SC is indicated in parentheses. Those oocytes in which the two foci were either touching or separated only by the width of an SC are considered synapsed, while those in which the GFP foci were well separated are scored as unsynapsed. Comparisons of these three methods of estimation are presented in Table 2.

**Table 2:**
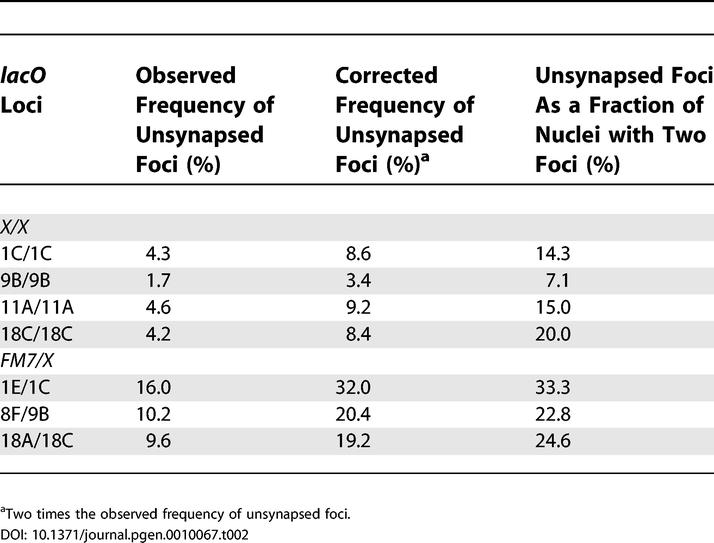
Summary of Synapsis Assays in *X/X* and *FM7/X* Oocytes

As an alternative to the simple qualitative characterization of two GFP foci as “synapsed or unsynapsed,” we also measured the distances between *lacO* sites in all oocytes in which we could clearly distinguish two GFP foci (even if they were overlapping). The histograms describing those distributions for *lacO* sites on normal sequence *X* chromosomes are presented in Figure 3A. Our analysis of *X/X* oocytes with two allelic *lacO* arrays revealed that those paired and synapsed foci that flanked a stretch of SC were never separated by more than 0.7 μm and were usually separated by only the width of the C(3)G signal that defines the SC ( ~0.4 μm). Thus, for purposes of the comparison of these data with the qualitative data on synapsis and non-synapsis presented in Table 1, those foci less than 0.7 μm apart may be considered as paired and synapsed and those foci separated by a distance of greater than 0.7 μm may be considered as unpaired or unsynapsed. Although distant or unpaired foci were rare in all four *X/X* genotypes studied, oocytes were occasionally observed in which either the 1C or 18C arrays were separated by distances substantially greater than 0.7 μm. While these observations demonstrate that most homologous sites are properly paired during meiosis, cases of failed pairing and synapsis do occur even in oocytes with iso-sequential *X* chromosomes.

### Examining Oocytes with *lacO* Arrays Located at Different Sites on Two Normal Sequence *X* Chromosomes

**Figure 4:**
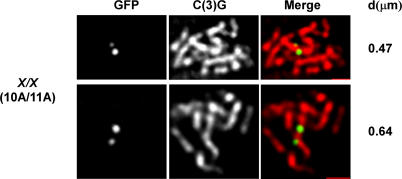
Synapsed and Unsynapsed *lacO* Sites in *X/X* (10A/11A) Oocytes Note that in the lower (unsynapsed) case, the two GFP foci are displaced along the length of the same stretch of SC. In these images, which consist of one to two optical sections, two GFP foci (green) are associated with a segment of SC (red). Distances between those GFP foci are shown at the right-most in each row. Bars = 1 μm.

The analysis presented above assumes that if two *lacO* arrays were frequently separated, even by small distances, we would still be able to visualize them as two separate dots. To estimate the effect of displacing two *lacO* arrays on the separation of GFP foci, we also assessed pairing and synapsis in *X/X* females carrying a *lacO* insertion at position 10A on one homolog and a *lacO* insertion at position 11A on the other. These two sites are separated by a physical distance of 0.9 Mb. Figure 4 presents examples of two nuclei in which the two foci were associated with a stretch of SC. In the upper case the two GFP foci were opposite from each other across the SC and scored as paired and synapsed, while in the lower case the two foci were well separated on the same stretch of SC and were considered to be unpaired. As shown in Table 1, among the 57 oocytes with two distinct GFP foci we saw 26 examples in which the two foci appeared as synapsed foci separated only by a stretch of SC. Among those 31 cases in which the two foci were not paired, 25 were nonetheless still associated with the same stretch of SC, and there were also six oocytes in which distant foci were not connected by SC. There were also 43 oocytes with just one GFP focus.

Figure 3B presents the distance distributions for the GFP foci in 10A/11A heterozygotes as well as distributions for two more distant pairs of *lacO* sites. These *lacO* insertions, which were also located on two normal sequence *X* chromosomes, are separated by physical distances of 1.6 Mb *(*11A/9B*)* and 11.7 Mb (12D/2F) (For comparison, the physical length of the *X* chromosome euchromatin is 22 Mb.). The distance distribution for the pair of allelic sites at 11A is presented for comparison. It is clear that as the distance between the *lacO* sites increases, so does the average distance between GFP foci.

### Assessing Chromosome Pairing in *X/X* Oocytes Homozygous for the *c(3)G* Mutation

**Figure 5:**
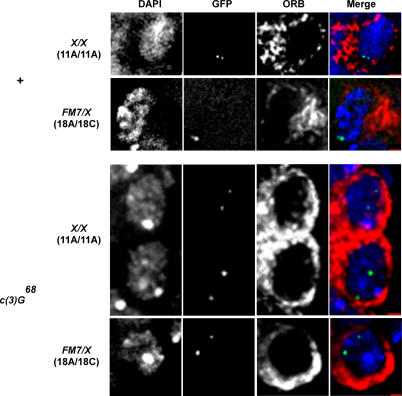
Unpaired *lacO* Sites in *c(3)G* Mutant Oocytes for Both *X/X* and *FM7/X* Oocytes Cytoplasmic protein marker ORB was used to identify the 16-cell cysts. It is present from region 2a, where it is evenly distributed in the 16 cells. At region 2b and region 3, ORB concentrates in the pro-oocytes and the oocyte. The GFP foci (green) in merge images in conjunction with DNA (blue, DAPI staining) and ORB (red). Two optical sections are shown in wildtype, while six to seven optical sections are shown in *c(3)G* mutants. Bars = 1 μm.

We were concerned that the associations of homologous chromosomes into regional domains within the nucleus might constrain both *X* chromosomes into a small enough nuclear region that we might fail to see unpaired *lacO* couplets even if they did occur. To confirm that high frequencies of failed pairing could be observed, if they indeed occurred, *lacO* pairing was analyzed in oocytes homozygous for the *c(3)G* mutation, which disrupts the pairing of euchromatic regions during zygotene/pachytene [1] (Figure 5). These experiments differ from those presented above only in that we used Orb staining to identify meiotic nuclei [23] rather than C(3)G itself. Examining oocytes homozygous for both a *lacO* insertion at 11A and for *c(3)G* revealed 15/33 nuclei with two unpaired foci. There were only three nuclei in which two distinct GFP foci were paired (for examples of unpaired foci in this genotype, see Figure 5). The distribution of distances between foci in oocytes with two GFP foci is shown in Figure 3C. By multiplying the frequency of unpaired foci by two or by computing the fraction of two foci nuclei that were unpaired (15/18), we can estimate that the *lacO* arrays were unpaired in 83%–91% of the oocytes examined. Thus, we can easily observe a failure in homolog pairing in *X*/*X* females that are homozygous for *c(3)G*.

### Use of the LacI-GFP: *lacO* System to Assess Chromosome Pairing and Synapsis in *Drosophila* Oocytes Heterozygous for the *FM7* Balancer Chromosome and a Normal Sequence *X* Chromosome

To obtain *lacO* sites in corresponding positions on *FM7*, we mobilized *lacO* arrays to multiple sites on the *FM7* chromosome and then chose those insertions that are located close to the positions of *lacO* sites on normal sequence *X* chromosomes. These include insertions at positions 1E, 8F, and 18A. By combining *FM7* chromosomes carrying these insertions with normal sequence *X* chromosomes carrying a *lacO* insertion at a nearby site, we created the following *lacO* couplets: a couplet at 1E *(FM7)/*1C*(X),* which defines a region just proximal to the distal break of *In(1)sc^8^* at 1B, a couplet at 8F*(FM7)/*9B*(X)* which defines a region within *In(1)dl-49*, and a couplet at 18A*(FM7)/*18C*(X),* which lies within *In(1)15DE-20AE*. The positions of these couplets in *FM7/X* heterozygotes are diagrammed in Figure 2. We compared the pairing and synapsis behavior of these three couplets of *lacO* arrays with the behavior of four allelic pairs of *lacO* arrays located at 1C, 9B, 11A, and 18C on a pair of normal sequence *X* chromosomes (see Table 1 and Figures 2 and 3).

The lower half of Table 1 displays the frequencies of failed pairing and synapsis for the three *lacO* couplets studied in *FM7/X* oocytes, as assayed by measuring the frequency of oocytes with two well-separated spots. Quite surprisingly, in most oocytes examined, these three couplets were paired and synapsed. Nonetheless, the observed frequencies of failed synapsis were higher than observed for *lacO* allelic pairs at similar positions in *X/X* females. Indeed, the observed frequencies of failed synapsis for the 8F/9B and 18A/18C *lacO* couplets were 10.2% and 9.6%, respectively. The frequency of unsynapsed foci for the 1E/1C couplet (16%) that lies close to the breakpoint of *In(1)sc^8^* at 1B was substantially higher than observed for the two couplets with breakpoints in the middle of the two smaller inversions. Using the correction of a factor of two required to compensate for the fact that each lacO site is detectable in only 70% of the oocytes suggests that actual frequencies of failed pairing lie between 19.2% and 32.0% (Table 2).

The corrected frequencies of failed synapsis presented in Table 2 correlate well with the frequencies of failed synapsis calculated by only using oocytes with two discernable GFP foci (see also Table 2). For example, in the case of the 1E/1C couplet we observed 60 nuclei in which the two foci were paired and straddled an intact region of SC (for examples, see Figure 2) and 30 cases in which the two foci were well separated, suggesting that the frequency of failed synapsis is 33.3%. For the 8F/9B couplet, 18 out of 79 nuclei with two GFP foci were unsynapsed (22.8%); and for 18A/18C, 16 out of 65 nuclei with two GFP foci were unsynapsed (24.6%). It is important to note that in *FM7/X* oocytes in those cases in which two GFP foci were scored as unsynapsed, the GFP foci were not observed to be connected by a stretch of SC. Thus, this situation is unlike the case described above for *X/X* oocytes that were doubly heterozygous for *lacO* insertions at 10A and 11A, in which even well-separated (unsynapsed) GFP foci were still found on opposite sides of a contiguous SC. Rather, these instances of unsynapsed foci in *FM7/X* oocytes appear to represent real cases of failed pairing and synapsis in the presence of the balancer chromosome, rather than simply the result of the distance between the two sites of *lacO* insertion on the *X* and *FM7* chromosomes. The histograms describing distributions of the distances between the foci in each of these genotypes are presented in Figure 3A, and no significant difference from those three allelic *lacO* pairs on *X/X* chromosomes was observed (for all three comparisons, *p* > 0.09).

We also note that the frequencies of failed synapsis are similar for oocytes in regions 2a, 2b, and 3 of *Drosophila* oocytes that correspond to the zygotene and pachytene stages of meiotic prophase (Table 1). The fact that the frequencies of failed synapsis are stable throughout meiotic prophase argues strongly that synaptic adjustment does not occur in *Drosophila* oocytes.

### High Frequencies of Failed Pairing in *FM7/X* Oocytes Can Be Induced by Homozygosity for *c(3)G*

The data presented above suggest that the three regions being assayed in *FM7/X* females are properly paired in approximately 70% of oocytes. However, given the dramatic exchange suppression observed in this genotype, we wondered whether or not we might have missed failed pairings in *FM7/X* heterozygotes for structural reasons. Perhaps the conformational twisting resulting from the need to maintain heterochromatic associations in the presence of heterozygosity for an inversion with heterochromatic breakpoints might restrict the *X* and *FM7* chromosomes to a small enough nuclear territory or domain that *lacO* foci might appear paired even in the absence of proper pairing. This possibility can be directly tested by examining *lacO* pairing in oocytes homozygous for the *c(3)G* mutation that causes a failure of pairing in euchromatic regions during zygotene/pachytene [1] without disrupting heterochromatic associations [21]. Examining *FM7/X* oocytes carrying the *lacO* couplet at 18A/18C and homozygous for *c(3)G* revealed 13/23 nuclei with two unpaired foci, one nucleus with two paired foci, and nine nuclei with a single GFP focus (for examples of paired and unpaired foci in this genotype, see Figure 5). The distribution of distances in oocytes with two GFP foci is shown in Figure 3C. By multiplying the frequency of unpaired foci by two or by computing the fraction of two foci nuclei that were unpaired (13/14), we can estimate that the *lacO* arrays were unpaired in 93%–100% of the oocytes examined. Thus, we can easily observe a failure in homolog pairing in *FM7/X* females that are homozygous for *c(3)G*.

In addition to the oocytes considered above, we also observed two oocytes that had four separated foci. A small number of oocytes with 3–4 FISH signals were also observed by Sherizen et al. [1] in *c(3)G* homozygotes. As suggested by those authors, the existence of these oocytes presumably reflects a role of the C(3)G protein in the maintenance of euchromatic sister chromatid cohesion as well as in the maintenance of homolog–homolog association. It is worth noting that both of these roles appear to be restricted to euchromatin; *c(3)G* oocytes show normal pairing in the heterochromatin [21].

### Comparing the Frequencies of Failed Pairing and Synapsis in *X/X* and *FM7/X* Oocytes

Table 2 compares the frequency of failed synapsis for allelic *lacO* sites in *X/X* females and *lacO* couplets in *FM7/X* females by the three metrics considered above (observed frequency of two separated foci, corrected frequency of separated foci, and fraction of oocytes with two foci in which the foci are unsynapsed). Although all regions tested appear to be paired and synapsed in the majority of oocytes of both genotypes, it is clear that the frequencies of failed synapses are higher in *FM7/X* heterozygotes. The highest frequency of failed synapsis in *FM7/X* oocytes (~33%) was observed for the 1E/1C *lacO* couplet. This region is of specific interest because in *FM7/X* oocytes these two *lacO* arrays lie immediately proximal to the distal breakpoint of *In(1)sc^8^*, a distance less than 3% of the length of the *X* euchromatin. The presence of proper pairing and synapsis in the remaining two-thirds of *FM7/X* oocytes argues strongly that breakpoints do not usually disrupt pairing and synapsis even in the immediate vicinity of the breakpoint. For the remaining two *lacO* couplets (8F/9B and 18A/18C), which define regions lying in the middle of *In(1)dl-49* and *In(1)15D-20AE*, the frequencies of apparent proper pairing and synapsis are estimated to be 75%–80%.

These observations argue strongly that the ability of the *FM7* balancer chromosome to suppress exchange by more than 100-fold when heterozygous cannot be explained by a corresponding strong suppression of pairing and/or synapsis. However, the types of observations presented here cannot exclude the possibility that there are subtle differences in homolog–homolog associations or synapsis in *FM7/X* heterozygotes, or defects in SC structure, that cannot be resolved by the techniques employed here. Nonetheless, as shown in the next two sections, if such differences do exist, they do not preclude the normal initiation of recombination as evidenced by DSB formation.

### *X* Chromosomes in *FM7/X* Heterozygotes Experience a Normal Number of DSBs

As noted in the Introduction, the *FM7* chromosome functions to suppress crossing over, rather than simply eliminating crossovers that do occur. Still, our failure to detect a defect in either pairing or synapsis makes the absence of crossing over in *FM7/X* heterozygotes difficult to understand. To further investigate the mechanism underlying the crossover suppression in *FM7/X* heterozygotes, we sought to determine whether the suppression could be the result of the prevention of DSB formation between *FM7* and the normal sequence *X* chromosome.

To determine whether DSBs were formed along the length of the synapsed *FM7/X* pair, we visualized sites of DSB formation using γ-HIS2AV antibody [24,25] as well as paired *lacO* sites in C(3)G-positive nuclei. Such an analysis requires finding only those cytologically favorable nuclei in which a well-separated length of SC is marked by paired GFP foci, and then assaying that stretch of SC for the presence of a γ-HIS2AV focus (or foci) indicative of DSB formation.

**Figure 6:**
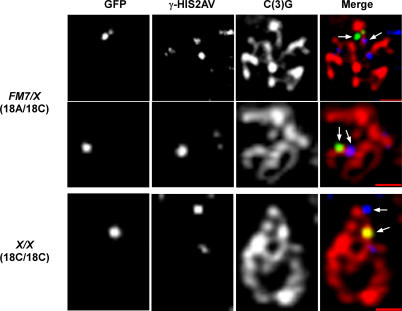
DSB Formation on the Same Stretch of SC As Paired *lacO* Couplets in *FM7/X* and *X/X* Oocytes The representatives from *FM7/X* (18A/18C) and *X/X* (18C/18C) oocytes are shown in this figure. Single C(3)G-positive (red) meiotic cells are shown in each row. DSBs are indicated by γ-HIS2AV staining (blue, arrows). GFP foci (green, arrows) represent the pairing *FM7* and *X* or *X* and *X* chromosomes. Two to three optical sections of images were projected. Bars = 1 μm.

In this study, allelic pairs at 9B and 18C in *X/X* females were compared with *lacO* couplets at 8F/9B and 18A/18C in *FM7/X* females. As shown in Figure 6 and Table 3, such cases of SC stretches doubly marked with GFP foci and a γ-HIS2AV focus were observed in ~5% of the SC-positive nuclei for all four genotypes studied. Indeed, combining the data for the two nearby *lacO* couplets studied in *FM7/X* oocytes, we observed 12 cases in which the same stretch of SC was marked by both a GFP focus (or paired foci) and by a γ-HIS2AV focus out of 237 oocytes examined in *FM7/X* oocytes (5.1%). Similarly (again combining the data for the two allelic pairs of *lacO* insertions studied in *X/X* females), nine cases in which the same stretch of SC was marked by both a GFP focus (or paired foci) and by a γ-HIS2AV focus were observed in 190 oocytes from *X/X* females (4.7%). Furthermore, the average distances between a GFP focus and a γ-HIS2AV focus were not significantly different between *FM7/X* and *X/X* oocytes (*p* = 0.86; the average distance in *X/X* is 0.79 μm while the value is 0.81 μm in *FM7/X*). While it is not possible to compare either of these frequencies to some absolute expectation of the number of such foci per given length of SC, it is clear that there is no obvious reduction in the frequency of DSBs in balancer heterozygotes.

**Table 3:**
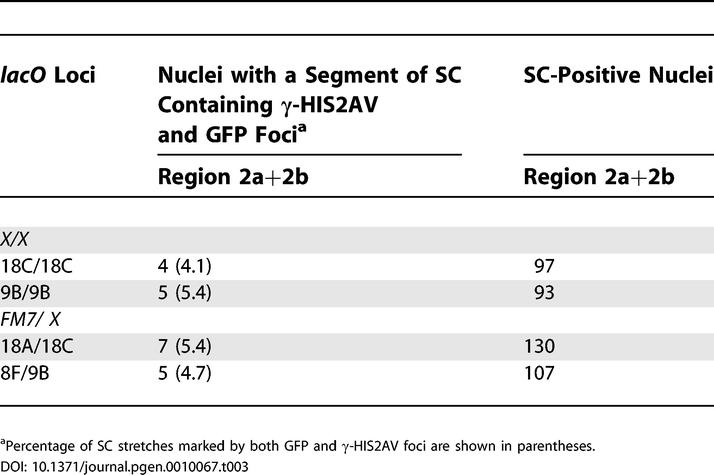
DSB Formation and Synapsed *lacO* Sites on the Same Stretch of SC

### Even a Global Suppression of Crossing Over Does Not Alter the Frequency of DSB Formation

Because of the difficulties inherent in finding cytologically favorable nuclei in which a well-separated length of C(3)G staining is marked by paired GFP foci, and then assaying that stretch of SC for the presence of a γ-HIS2AV focus, we chose to simply measure the number of DSBs occurring in oocytes in which exchange is suppressed on all five chromosome arms as a result of heterozygosity for three balancer chromosomes *FM7, SM1*, and *TM3*. The *SM1* and *TM3* balancer chromosomes each involve six euchromatic breakpoints. They also strongly suppress exchange when heterozygous with normal sequence homologs, as evidenced by their sensitivity to nondisjunction induced by mutants that impair the achiasmate segregation systems [26,27].

**Figure 7:**
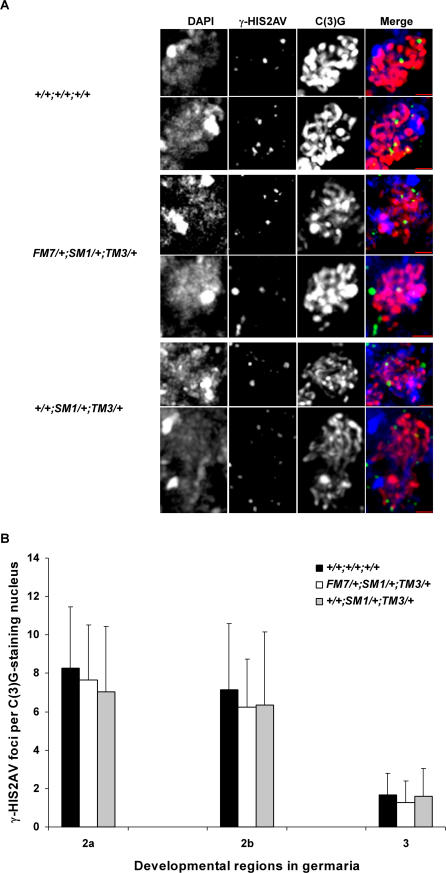
DSBs in Oocytes Heterozygous for Three Balancer Chromosomes (A) Representatives from the oocytes with normal or heterozygous balancer chromosomes. Maximum intensity projection of image stacks of nuclei showing C(3)G (red) in conjunction with DNA (blue, DAPI staining) and DSB (green, γ-HIS2AV staining). Bars = 1 μm. (B) Distribution graph representing the number of γ-HIS2AV foci per C(3)G staining nucleus at different developmental regions in germaria. The sample size of oocytes in wildtype is 64, 34, and 9, representing region 2a, 2b, and 3; in *FM7/+;SM1/+;TM3/+*, 48, 43, and 11; in *+/+;SM1/+;TM3/+*, 66, 38, and 10, respectively. Data are presented as means ± SD.

Data for both γ-HIS2AV staining and C(3)G staining in oocytes doubly or triply heterozygous for these balancers are presented in Figure 7. We saw no obvious difference in either the number of γ-HIS2AV foci per nucleus during the length of meiotic prophase or in the general structure or organization of the SC when comparing wildtype oocytes. This observation suggests that even when confronted with two or three balancer chromosomes, both extensive synapsis and DSB formation still occur in *Drosophila* oocytes. Moreover, this experiment also suggests that the well-documented ability of heterozygous inversions to increase the frequency of recombination elsewhere in the genome, referred to as the “interchromosomal effect” [28], is not mediated by either a substantial increase in the total number of DSBs or by an obvious change in the timing of their appearance or disappearance.

### Effects of the *FM7* Balancer Chromosome on Pre-Meiotic Pairing Are Similar to Its Effects on Meiotic Pairing

Table 4 presents data for pre-meiotic pairing of allelic lacO insertion sites in *X/X* germlines and of nearby lacO couplets in *FM7/X* germlines. These interphase nuclei were obtained from regions 1 and 2a of the germarium and include mitotically dividing cystoblast and cystocyte cells (region 1) as well as nuclei from 16 cell cysts that have not yet assembled SC (region 2a). For *X/X* nuclei, the frequencies of failed pairing (as indicated by two well-separated GFP foci) ranged from 1.4 % (1C) to 6.4 % (18C). While the frequencies of failed pairings were higher for the *FM7/X* lacO couplets (5.9% to 18.3%), they are still substantially less than frequency of unpaired GFP foci (39.2%, *n* = 51) observed in pre-meiotic nuclei in *X/X* females carrying a lacO insertion at position 10A on one homolog and a lacO insertion at position 11A on the other (a physical distance of 0.9 Mb). The observation that the effects of *FM7* on pre-meiotic pairing are similar to its effects on meiotic pairing support an emerging view that homolog pairing relationships are established early in *Drosophila* development and maintained until the completion of synapsis during meiosis [1,2]. These data also confirm and extend the preliminary cytological observations of Becker [29] that suggested that at least for the large *In(1)sc8* inversion, the sequences within the inversion can pair with a normal sequence homolog in somatic cells.

**Table 4:**
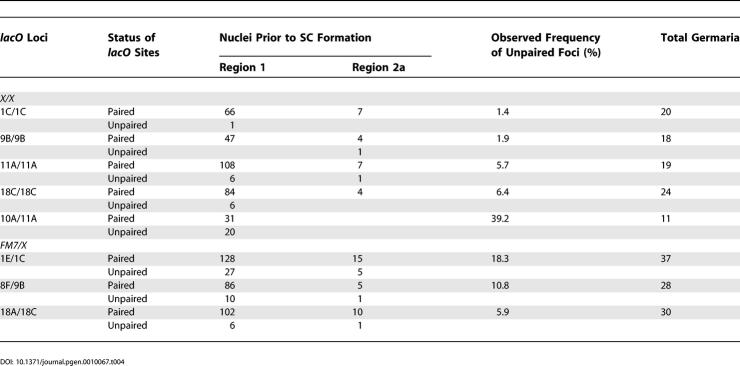
Chromosome Pairing As Assayed by LacI-GFP Tagging

## Discussion

The data presented above argue that while the oocytes heterozygous for the *FM7* balancer chromosome and for a normal sequence *X* chromosome do exhibit a higher frequency of failed pairing and synapsis than do oocytes carrying two normal sequence *X* chromosomes, the effect is small in comparison to the global defect in exchange observed in *FM7/X* females. Moreover, the fact that the frequencies of failed pairing and synapsis do not increase throughout meiotic prophase argues strongly that synaptic adjustment does not occur in *Drosophila* oocytes. In that sense, our data confirm and extend the studies of translocation heterozygotes performed by Sherizen et al. [1] and demonstrate that despite their ability to suppress exchange over large distances, heterozygous breakpoints do not create corresponding strong defects in pairing or synapsis. However, we do see at least a weak defect in pairing that appears to be strongest in the interval closest to a breakpoint. The possible significance of these defects is discussed below. Finally, our data also allow us to conclude that the exchange suppression generated by breakpoint heterozygosity is not the result of a strong decrease in the frequency of DSBs.

While we observed that *X* chromosomal bivalents are paired and synapsed in the large majority of *FM7/X* oocytes, we nonetheless do observe an increased frequency of failed pairing and synapsis in *FM7/X* heterozygotes. The effect on pairing and synapsis that we see may parallel an effect on synapsis in the vicinity of the breakpoints in translocation heterozygotes observed by Sherizen et al. [1]. These authors, who used FISH to analyze pairing and synapsis near the breakpoints, observed that the SC staining in translocation heterozygotes was sometimes less intense than was observed in wildtype controls and was missing entirely in 10%–20% of nuclei. Although we do not see a decrease in C(3)G intensity in *FM7/X* heterozygotes, the frequency of synapsis failures observed by Sherizen et al. [1] (10%–20%) is roughly similar to the frequencies of failed pairing and synapsis observed in our study. Thus, in both inversion and translocation heterozygotes, it appears that pairing and synapsis still occurs between the rearrangement and the normal sequence homolog in the majority of oocytes.

### How Might Heterozygosity for a Breakpoint Suppress Exchange, without Suppressing Pairing and Synapsis in *Drosophila*?

Several aspects of its meiotic process make the *Drosophila* oocyte different from meiotic cells in many other species. First, in both male and female meiosis, *Drosophila* homologs either enter meiosis in a fashion that preserves existing pre-meiotic pairings [1,2, and this study], or they are able to rapidly establish pairing following cell division. Second, although the maturation of such pairings to synapsis via SC assembly does not require the formation of DSBs [6], DSB formation does occur in the absence of synapsis [25]. Third, the maturation of the DSBs to either reciprocal crossover events or gene conversion events absolutely requires the presence of the C(3)G Zip1-like protein [30,31]. Breaks that occur in the absence of C(3)G function are evidently repaired by conversion-like events involving either the sister chromatid or the homolog, because only slight, if any, increases in the frequency of sister chromatid exchange are observed in females homozygous for null mutants in *c(3)G* [6,32]. However, Carlson has presented evidence that inter-homolog conversion events are quite rare, if they occur at all [30].

Within the context of this meiotic system, we can propose two general classes of models to explain the ability of breakpoints to suppress exchange over relatively large distances, without obviously affecting pairing. According to the first model, proposed by both Hawley [33] and more fully by Sherizen et al. [1], the conversion of DSBs into crossovers would require a long region of uninterrupted continuity of the SC. Either the absence of that continuity or a structural change in the SC at the site of a breakpoint would serve to suppress crossover formation over long distances from the breakpoint.

It is possible that breakpoints might suppress exchange by a mechanism that is functionally similar to crossover interference. Indeed, the process of synapsis across a breakpoint might require a distortion or twisting of chromosome axes as they switch from pairing with one chromosome to the other (in the case of a translocation heterozygote), or from one chromosomal region to another (in the case of an inversion heterozygote), that mimics the effect of an actual crossover event on axis structure, and, in doing so, propagates a signal along the length of the SC that diminishes the likelihood of an actual exchange. This model has the intriguing feature that it explains the observation that the ability of breakpoints to suppress exchange in an organism with strong interference, such as *Drosophila,* is quite robust compared with their ability to suppress exchange in *Saccharomyces cerevisiae,* in which interference is weak [34]. It also explains the well-documented observation that genetic or environmental factors that reduce the level of interference (such as heat, age, the inter-chromosomal effect, or heterozygosity for *c(3)G*) significantly elevate the levels of recombination in regions suppressed by breakpoint heterozygosity. Finally, this model makes the prediction that heterozygous breakpoints should be much poorer suppressors of exchange in the closely related species *D. mauritiana,* in which interference is weak or absent [35], than they are in *D. melanogaster*.

According to the second model, one could imagine that breakpoints do create subtle or short-lived defects in pairing and synapsis that propagate over long distances and are not observable by current methods. While such “invisible defects” are, of course, hard to prove or disprove, their existence might explain the observations of Sherizen et al. [1] that both reciprocal recombination and gene conversion are suppressed by breakpoint heterozygosity. Given that exchange, and apparently gene conversion as well, requires proper synapsis in *Drosophila* (or at least the presence of the C(3)G protein) [30,31], a subtle defect in synapsis propagated over long distances might explain both defects. One mechanistic view of this model might take the form of proposing that there is a critical period early during zygotene/pachytene in which either C(3)G must be present and/or SC must properly form in order to allow DSBs to be properly matured to either reciprocal exchanges or gene conversion events. If breakpoint heterozygosity delayed C(3)G action or incorporation within or beyond that brief temporal window, one might see dramatic reductions in exchange. The subsequent proper assembly of the SC might well mask such a brief and early defect. This model has the benefit that with development of the technology to visualize meiotic prophase in living oocytes, it might eventually be testable. An alternative version of this model might suggest that the pairing and synaptic failures that occur close to a breakpoint result in a propagated disruption in SC structure that, while too subtle to be observed by the techniques proposed here, is nonetheless sufficient to prevent the maturation of DSBs into crossovers.

Both of these models allow us to propose roles for the crossover-suppression boundary sites mapped by both Hawley [33] and Sherizen et al. [1]. While it now seems unlikely that such sites play a role in mediating meiotic pairing, it seems likely that they define regions in which proper synapsis or SC structure can be restored in such a way that the effects of a heterozygous breakpoint on synapsis or SC structure are damped out across the lengths of these regions. Such a function may well be consistent with the finding that at least the sites mapped by Hawley [33] reside in regions of intercalary heterochromatin. Such a role for these sites as “fasteners” of synapsis is consistent with the proposal of Sherizen et al. [1] that these sites are involved in defining large chromosomal domains that control crossover formation, perhaps by playing roles in either initiating SC formation or correcting deformations in SC structure.

Two other possibilities, which we deem less likely, also need to be at least mentioned. The first is that chromosomes that dominantly suppress the recovery of crossovers may well acquire a significant amount of sequence divergence as a consequence of reduced recombination. This may be especially true for balancer chromosomes such as *FM7* that suppress exchange along their entire length. One could imagine that the accumulation of such sequence divergence might suppress the formation of those recombinational intermediates that facilitate both reciprocal exchange and gene conversion. While this model is attractive in terms of simplicity, it fails to explain how a translocation breakpoint might suppress exchange. Moreover, our limited amount of sequence analysis on the *FM7* balancer chromosome, performed in the vicinity of the *Axs* locus, suggests a relatively low level of sequence polymorphism (~2%) over a 2-kb region (Gustafson and Hawley, unpublished data). This level of polymorphism is unlikely to greatly reduce the rate of recombination [36]. Finally, Coyne and his collaborators have characterized one pericentric inversion that is coupled to a cis-acting mutant that dominantly suppresses exchange within the inverted region [37]. While such mutants clearly exist, the fact that most aberrations (including *FM7*) allow normal, or near normal, levels of exchange when homozygous renders this possibility unlikely.

### What Is the Fate of DSBs in Balancer Heterozygotes?

Given that the DSBs that occur along the length of the *FM7/X* bivalent are not matured into crossovers, it becomes important to understand just how they might be repaired. Studies of exchange in females heterozygous for both *FM7* and a ring-X chromosome (*FM7*/*R(1)* females) presented in McKim et al. [6] failed to show a substantial level of ring chromosome loss, as might be expected if DSBs were frequently processed to sister chromatid exchanges [38]. One possibility is that these events are repaired by either inter-homolog gene conversion events or by gene conversion events involving the sister chromatid. The effect of breakpoint heterozygosity on conversion is unclear. Sherizen et al. [1] found a greater than 6-fold reduction of inter-homolog conversion events near a breakpoint in translocation heterozygotes. Perhaps then, in the vicinity of the breakpoint, sister chromatid conversion events predominate, while at greater distances from the breakpoint repair by inter-homolog conversion events becomes more frequent.

However, Chovnick [39] found little or no effect on gene conversion when comparing conversion at the *rosy* locus in females with two normal sequence third chromosomes and in females heterozygous for a paracentric inversion that includes *rosy*. One possible explanation for this discrepancy might lie in the fact that the two breakpoints studied by Sherizen et al. [1] were within one numbered polytene division of the *rosy* locus, while both breakpoints of the inversion studied by Chovnick [39] were greater than four polytene units away from *rosy*. Indeed, the inversion studied by Chovnick [39] included two “boundary or pairing site” elements (see [1]) that might function to restore pairing and synapsis.

### Heterozygosity for Aberration Breakpoints and Exchange Suppression in Other Species

Numerous meiotic systems have been characterized in which breakpoint heterozygosity leads to absent or aberrant synapsis. As reviewed by Koehler et al. [40], studies of pairing and synapsis of simple inversion heterozygotes in various organisms have revealed three major patterns of pairing and synapsis. In the first pattern, *homologous synapsis,* the sequences within the inversion pair and synapse properly with their homolog. In the second process, *synaptic adjustment,* the inversion loop that is initially formed by homologous pairing is gradually re-adjusted by progressive heterologous synapsis until the loop has been replaced by a linear stretch of SC running from end to end of the bivalent. In the third process, *heterologous synapsis,* no loop is formed and heterologous synapsis occurs concomitantly with the establishment of homologous synapsis elsewhere in the genome. Although both synaptic adjustment and heterologous synapsis are well documented, organisms, and even individual aberrations, appear to differ in terms of which process predominates (for review, see Koehler et al. [40]). Our data suggest that the frequency of pairing at the three sites we monitored does not change during meiotic prophase. Indeed, the frequencies of failed pairing in SC positive cells are quite similar to those observed in pre-meiotic nuclei. Thus, it seems unlikely that a process analogous to synaptic adjustment occurs in *Drosophila* oocytes.

## Materials and Methods

### *Drosophila* strains.

The transgenic construct expressing *LacI-GFP* and the autosome *lacO* transgenic lines were gifts from A. S. Belmont and J. W. Sedat, and described in Vazquez et al. [2]. We then moved the *lacO P*-element insertion from sites on the autosomes to new locations on normal *X* and *FM7* chromosomes by providing the transposase source Δ2–3. The positions of the new *lacO* insertions were subsequently determined by inverse PCR. Genomic DNA was digested by Sau3AI or MspI; after ligation, PCR was performed using primer pairs CGGATATATGTCGGCTACTCCTTGC and CACCCAAGGCTCTGCTCCCACAAT, or CTAGGTACGGCATCTGCGTTGAGTC and ATTGAGACGAAATGAACCACTCGGA. *c(3)G^68^* mutant lines were described in [41].

### Antibodies and immunofluorescence.

All the immunolocalization experiments were carried out as described in [41]. The mouse anti-C(3)G antibody [41] was used at 1:500; both mouse monoclonal Orb antibodies 4H8 and 6H4 [23] were used together at 1:30. Secondary antibodies Cy3-conjugated (Jackson ImmunoResearch, West Grove, Pennsylvania, United States) and Alexa 647-conjugated (Molecular Probes, Eugene, Oregon, United States) anti-mouse IgG were used at a dilution of 1:500.

For detecting DSBs, the anti-phospho-H2AV(Ser139) rabbit polyclonal antibody (Upstate Biotechnology, Lake Placid, New York, United States) was used at 1:100, and the secondary antibody Cy3-conjugated anti-rabbit (Jackson ImmunoResearch) was used at 1:300. All distinct γ-HIS2AV foci were counted, regardless of the size of the individual. The size distribution of γ-HIS2AV foci appeared to be comparable in all the different genotypes of flies.

### Microscopy and image analyses.

Images were collected using a DeltaVision microscopy system (Applied Precision, Issaquah, Washington, United States), equipped with an Olympus IX70 inverted microscope and high-resolution CCD camera. Image data were deconvolved using the softWoRx v. 2.5 software package (Applied Precision). The distance between the centers of two GFP foci was measured using the same software.

### Identifying oocytes within the germarium.

In the *Drosophila* ovariole, germline stem cells are located at the anterior-most tip of the germarium. Following stem cell division, primary oogonial cells (cystoblasts) undergo four mitotic divisions with incomplete cytokinesis to create 16 cell cysts in which the cells are inter-connected by ring canals. We identified meiotic cells by staining for the C(3)G protein, which comprises the transverse filaments of the SC. In region 2a, two to four cells of each ball-shaped 16-cell cyst enter meiosis (as identified by SC formation). After this point, cysts move toward the posterior of the germarium, where they first flatten out to a pancake-like shape (region 2b) and then are enveloped by a monolayer of follicle cells at the posterior end of the germarium (region 3).

### Assessing the frequency of pairing in meiotic and mitotic cells.

Our initial analysis of chromosome pairing and synapsis in *Drosophila* oocytes focused on the study of four allelic pairs of *lacO* arrays located at 1C, 9B, 11A, and 18C on a pair of normal sequence *X* chromosomes (see Table 1 and Figures 2 and 3). In its simplest form, such an analysis would look at each oocyte and determine whether or not the two *lacO* sites were paired, as evidenced by either the presence of a single GFP focus or two nearby foci that flanked a SC, or unpaired, as evidenced by two well-separated *lacO* foci. Unfortunately, such a simple type of analysis presumes that each *lacO* array is always detected in 100% of the oocytes. However, an analysis of oocytes that were heterozygous for a single *lacO* array showed that any given *lacO* array is visible in only approximately 70% of the oocytes. For three *lacO* arrays located on normal sequence *X* chromosomes at positions 1C, 9B, and 18C, the frequencies of oocytes carrying a single copy of this array that exhibited the expected single GFP focus were 65.3 % (*n* = 118), 72.8% (*n* = 114), and 68.0% (*n* = 103), respectively. Similarly, with three *lacO* arrays located on the *FM7* balancer chromosome at positions 1E, 8F, and 18A, the frequencies of oocytes carrying a single copy of this array that exhibited the expected single GFP focus were 74.3 % (*n* = 101), 65.4 % (*n* = 81), and 71.0% (*n* = 107), respectively.

Our ability to detect a single lacO array in only ~70% of the cases suggests that two well-separated *lacO* foci would be detectable in only 49% of the instances in which they occurred. Indeed, when we examined females that were doubly heterozygous for *lacO* foci located at distant sites along the X chromosome, the frequency of oocytes that exhibited the expected two foci was indeed approximately 50%. As expected, in the case of the 10A/11A double heterozygotes in *X/X* females, we observed only two discrete foci in 57% (*n* = 100) of the oocytes examined. Similar frequencies of nuclei with two GFP foci were also observed in females doubly heterozygous for *lacO* insertions at 9B and 11A (52.8%, *n* = 89) and for insertions at 2F and 12D (66.2%, *n* = 77). These observations suggest that we may miss one of the two *lacO* arrays in approximately 50% of oocytes. For this reason, our estimates of the frequency of failed pairing and synapsis may be under-estimated by as much as 2-fold, suggesting that the frequency of synapsis failure may actually range from 3%–10% for the four sites examined (Table 2). Similar observations were made in *FM7/X* oocytes that were doubly heterozygous for *lacO* insertions at 18A and 9B and at 8F and 18C. In the case of the 9B/18A double heterozygote, we were able to visualize two foci in 47.4% (*n* = 78) of the oocytes, while in 8F/18C double heterozygotes we were able to visualize two foci in 57.1% (*n* = 91) of the oocytes. These observations suggest that, as was the case for the *lacO* insertions studied in *X/X* females, we may be underestimating the frequency of failed pairing and/or synapsis by as much as a factor of two.

For these reasons, we have evaluated the frequency of failed pairing by three separate parameters. First, as exemplified in Table 1, we simply report the fraction of oocyte with two clearly separate GFP foci. While this metric is clearly an underestimate, it nonetheless can be used to compare the severity of pairing failures between the genotypes examined. Second, we provide a more accurate measurement of pairing failure by multiplying the observed fraction of oocytes with unpaired *lacO* foci by a factor of two. Finally, we obviate the detection problem by considering only that subset of oocytes that exhibit two discernable foci. Those oocytes in which the two foci were either touching or separated only by the width of an SC are considered paired, while those in which the GFP foci were well separated are viewed as unpaired. This approach also allows us to provide more quantitative estimates of the frequency of failed pairing by measuring the distances between the two foci in each oocyte. Comparisons of these three methods of estimation are presented in Table 2.

Finally, we were concerned that our frequency of failed pairing might be over-estimated by cases in which two foci were created by sister chromatid separation. We can discount this possibility for three reasons. First, because each *lacO* array has a characteristic intensity we could tell the difference between two unpaired arrays and two separated sisters. Second, as indicated by their absence in Table 1, we did not see oocytes with the three or four foci that might be expected if sister separation was common. We did however, see such oocytes (those with three to four dots) in c(3)G oocytes, confirming the observations of Sherizen et al. [1]. Third, we examined *lacO*:LacI-GFP interactions in oocytes with but one copy of the *lacO* array. In such oocytes, sister separation could be easily detected but the frequency of such events was extremely low. A discernable separation of nearby or overlapping foci was observed in 1%–3% of oocytes. For all of these reasons, it seems very unlikely that our estimates of the frequency of failed pairing might be greatly over-estimated due to sister chromatid separation.

### Statistics.

Statistical analyses were performed using Graphpad Instat software for Macintosh. The distance distributions were analyzed using the Mann-Whitney test. For statistical analyses of the distances of GFP foci (Figure 3A), the distance of overlapping foci was assigned as 0.125 μm.

## Abbreviations

DSB: double-strand break
GFP: green fluorescent protein
SC: synaptonemal complex
